# Dr. Wilson's Treatise on Febrile Diseases

**Published:** 1801-10

**Authors:** 


### CRITICAL RETROSPECT
### OF
### MEDICAL AND PHYSICAL LITERATURE.

---

### *Dr. Wilson's Treatise on Febrile Diseases.*

( Concluded from p. 271 of our last. )

In our last we gave an enumeration of the usual symptoms of the plague, omitting, however, several accidental variations of less importance. On the *Cause*, or manner of propagating the plague, Dr. W. says, " It will be unnecessary here to make many observations on contagion, as this subject has already been considered at length. It is needless to repeat what has been said of the means of preventing the generation and checking the progress of contagious diseases;* and it would be tedious here to describe lazarettos and the various precautions employed to prevent the introduction of the plague from foreign countries; for these I shall refer the reader to the works of Mr. Howard and Dr. Russell.

" Some facts would lead us to suppose that peculiar states of the air are favourable to the production of the plague; it has sometimes appeared in many parts of a country at the same time. More, generally, however, it appears at first in one place, and spreads gradually to others.

" Like other contagious fevers, warm weather is generally favourable, and cold weather unfavourable, to its progress. The plague was greatly weakened, De Mertens informs us, while the thermometer stood between sixteen and twenty degrees below frost. It often happens, however, that its violence is not checked by the winter, and there are many instances of the plague ceasing in the warmest seasons of the year. In all the plagues with which Aleppo has been visited during this century, the Rev. Mr. Dawes observes, it is said to have regularly and constantly ceased in August or September, the hottest months of the year.

" Like other contagions, that of the plague is active only for a very short distance around the patient. The physicians, De Mertens observes, often went within a foot of the patients, without being infected.

" The young and robust, the same author observes, were more

---

* De Mertens recommends, as a good preventive, wearing a cloak of oiled cloth over the clothes, while in the rooms of the sick. It has also been recommended to anoint the body with oil. De Mertens thinks issues not to be depended on. The reader will find, in his Treatise on the Plague of Moscow, formulæ for what have been termed anti-pestilential powders; they are similar to the fumigating powders mentioned in the first volume, when speaking of the purification of fomites; the manner of using them also, is there pointed out.

liable to infection than the old and infirm. It has often been remarked of the plague, as of most other contagious fevers, that infants are less liable to it than adults. Waldshmidt saw several infants who sucked nurses ill of the plague, and yet escaped infection. He even observes, that one infant sucked two nurses ill of the plague, both of whom died, yet the child did not receive the disease. A woman, who suckled her child five months old, was seized with the plague, and died after a week's illness, but the child who sucked her and lay in the same bed with her, escaped the distemper. De Mertens and others make the same observation.

It is remarkable, however, that the fœtus in utero sometimes receives the disease, and that even where the mother escapes. 'Last year as well as this,' says Mr. Dawes, 'there has been more 'than one instance of a woman's being delivered of an infected 'child with the plague sores on its body, though the mother her- 'self has been entirely free from the distemper.'*

"It was observed of some of the other exanthemata, that certain constitutions are incapable of undergoing them; the same seems to be true of the plague. Waldshmidt knew a woman who was servant in a family where seven people died of the plague, and afterwards in another, all of whom died of it, and yet remained uninfected. A Greek lad, Mr. Dawes observes, made it his business for many months to wait on the sick, and to wash, dress, and bury the dead, yet escaped the disease.

"Those who have once had the plague are less subject to it than others. This circumstance, together with the success which had attended inoculation for the small-pox, induced some to recommend inoculating for the plague, which has actually been done. In Dr. Guthrie's Letter to Dr. Duncan, there is an account of a surgeon, Mathias Degio, who inoculated himself for the plague. He inserted with a lancet, under the cuticle of the arm, a little of the matter from a pestilential abscess. On the fourth day after inoculation the fever appeared; he treated himself in all respects in the same way as if he had been inoculated for the small-pox, paying particular attention to the cool regimen. His only medicines were cold water and vinegar, with a little wine. The complaint proved so mild, that he was never confined to bed, but was generally in the open air. It was not a more severe complaint than the common inoculated small-pox.

"This surgeon afterwards regularly attended an hospital allotted for the reception of patients under the plague, without feeling any symptom of the disorder; while most of the other surgeons fell a sacrifice to it. From one case, however, nothing conclusive can be drawn; besides, it is far from being uncommon for the plague to attack the same person a second or third time."

On the *Treatment* our author agrees with Dr. Cullen, "the indications are the same as those of fever in general," or, perhaps, more particularly, the contagious typhoid fevers of large cities.

MEDICAL

* In pregnant women the plague generally produces abortion.

